# Metabolic syndrome, left ventricular diastolic dysfunction and heart failure with preserved ejective fraction

**DOI:** 10.3389/fendo.2025.1544908

**Published:** 2025-04-14

**Authors:** Dan Zhou, Shuisheng Lin, Zongchen Liu, Jiawen Yuan, Huixia Ren, Huiying Tan, Yi Guo, Xin Jiang

**Affiliations:** ^1^ Department of Geriatrics, Shenzhen People’s Hospital (The Second Clinical Medical College, Jinan University; The First Affiliated Hospital, Southern University of Science and Technology; Shenzhen Clinical Research Center for Geriatrics; Guangdong Provincial Clinical Research Center for Geriatrics), Shenzhen, Guangdong, China; ^2^ Department of Neurology, Shenzhen People’s Hospital, Shenzhen, Guangdong, China

**Keywords:** metabolic syndrome, left ventricular diastolic dysfunction, heart failure with preserved ejective fraction, hypertension, diabetes mellitus

## Abstract

Metabolic syndrome (MetS) encompasses a cluster of interrelated conditions, including obesity, hyperglycemia, hyperlipidemia, and hypertension, and has been established as a significant risk factor for cardiovascular events and heightened mortality. At its core, insulin resistance serves as the primary underlying mechanism driving the development of MetS. The prevalence of MetS is rising at an alarming rate, posing a significant public health challenge worldwide. Even in the absence of overt obstructive coronary artery disease or valvular heart disease, patients with MetS often exhibit adverse cardiac remodeling and myocardial dysfunction. Left ventricular hypertrophy (LVH) and left ventricular diastolic dysfunction (LVDD) are the leading manifestations of heart failure with preserved ejection fraction (HFpEF). Abnormal myocardial substrate utilization, neurohormonal activation, interstitial fibrosis, coronary microvascular dysfunction, and metabolic inflammation have all been implicated in the development and progression of adverse cardiac remodeling associated with MetS. However, despite the tremendous research produced on this subject, HFpEF remains highly prevalent in such a population. The early diagnosis of abnormal cardiac remodeling would enable optimal effective therapies to prevent the progression of the disease to the symptomatic phase. HFpEF encompasses a diverse range of pathological processes. In these patients, LVDD and elevated left ventricular filling pressure are the primary manifestations. Echocardiography remains the popular imaging modality for the assessment of LVDD and LV filling pressure. The article aims to review recent articles covering the association between MetS components or MetS and LVDD in HFpEF.

## 1 Introduction

Metabolic syndrome (MetS) appears to be important in the development of left ventricular diastolic dysfunction (LVDD) and its progression towards heart failure with preserved ejection fraction (HFpEF), as the increasing prevalence of obesity, hypertension, chronic kidney disease, and diabetes is closely associated with the incidence of HFpEF. HFpEF is commonly defined as a condition in which a person with heart failure (HF) has a left ventricular (LV) ejection fraction (EF) of ≥50%. The European Society of Cardiology (ESC) provides a more detailed definition: HFpEF is characterized by symptoms and signs of heart failure, evidence of structural and/or functional cardiac abnormalities, and/or elevated natriuretic peptides (NPs), along with an LVEF of ≥50% (1). Findings from previous studies have suggested a novel paradigm. In the new paradigm, multiple comorbidities, including obesity, hypertension, hyperlipidemia, diabetes, and insulin resistance, cause a systemic pro-inflammatory state that leads to oxidative stress, endothelial dysfunction, ectopic fat accumulation, and coronary microvascular dysfunction. Eventually, these disease mechanisms contribute to LVDD and, ultimately, HFpEF.

Throughout this review, we will focus on the association between MetS and its components with changes in LVDD to offer updated information on this emerging issue. Using the following keywords, we searched PubMed, Medline, OVID, and EMBASE databases for English-language studies published from January 1963 to December 2024: “left ventricle diastolic dysfunction”, “metabolic syndrome”, “arterial hypertension”, “systemic hypertension”, “obesity”, “overweight”, “central obesity”, “body mass index”, “diabetes”, “increased glucose level”, “dyslipidemia”, “triglycerides”, and “high-density lipoprotein cholesterol”, and “heart failure with preserved ejective fraction”.

## 2 Definitions of MetS

There have been several definitions proposed for the MetS so that clinicians and researchers can use the tool effectively. An international consultation group on the definition of diabetes for the World Health Organization (WHO) proposed the first formalized definition of metabolic syndrome in 1998 (2). Several insulin resistance markers and two additional risk factors, including obesity, hypertension, high triglyceride level, reduced high-density lipoprotein (HDL) cholesterol level, or microalbuminuria, could be used to diagnose the syndrome by the WHO criteria. Based on the National Cholesterol Education Program-Third Adult Treatment Panel (NCEP ATP III) definition (3), a diagnosis is based on the presence of three of the following five factors: abdominal obesity, elevated triglyceride, reduced HDL cholesterol, elevated blood pressure (BP), and elevated fasting glucose (impaired fasting glucose or diabetes). In 2005, according to the International Diabetes Federation (IDF) (4), abdominal obesity was one of five factors that must be taken into account in the diagnosis of diabetes, with waist measurement serving as the primary screening tool. The remaining criteria were essentially the same as those in ATP III. While the American Heart Association (AHA)/National Heart, Lung, and Blood Institute (AHA-NHLBI) definition (5) slightly modified the ATP III criteria, abdominal obesity was not mandated as a risk factor. In 2009 (6), both the IDF and AHA/NHLBI recognized abdominal obesity as one of five criteria for a diagnosis, but not a prerequisite. As a result, the presence of three of five risk factors constitutes a diagnosis of MetS. **Table 1** shows the common definition.

**Table 1:** Criteria for the clinical diagnosis of metabolic syndrome.

Measure	Categorical cut-off points
Elevated waist circumference*	Population- and country-specific definitions
Elevated triglycerides (drug treatment for elevated triglycerides is an alternate indicator**†**)	≥150 mg/dL (1.7 mmol/L)
Reduced HDL-C (drug treatment for reduced HDL-C is an alternate indicator**†**)	<40 mg/dL (1.0 mmol/L) in men; <50 mg/dL (1.3 mmol/L) in women
Elevated blood pressure (antihypertensive drug treatment in a patient with a history of hypertension is an alternate indicator)	Systolic ≥130 and/or diastolic ≥85 mm Hg
Elevated fasting glucose**‡** (drug treatment of elevated glucose is an alternate indicator)	100 mg/dL

HDL-C, high-density lipoprotein cholesterol.

*It is recommended that the IDF cut-off points be used for non-Europeans and either the IDF or AHA/NHLBI cut-off points be used for people of European origin until more data are available.

†The most commonly used drugs for elevated triglycerides and reduced HDL-C are fibrates and nicotinic acid. A patient taking one of these drugs can be presumed to have high triglycerides and low HDL-C. High-dose Omega-3 fatty acids presumes high triglycerides.

**‡**Most patients with type 2 diabetes mellitus will have the metabolic syndrome by the proposed criteria.

## 3 Echocardiographic variables for evaluating LVDD

**Figure 1:**
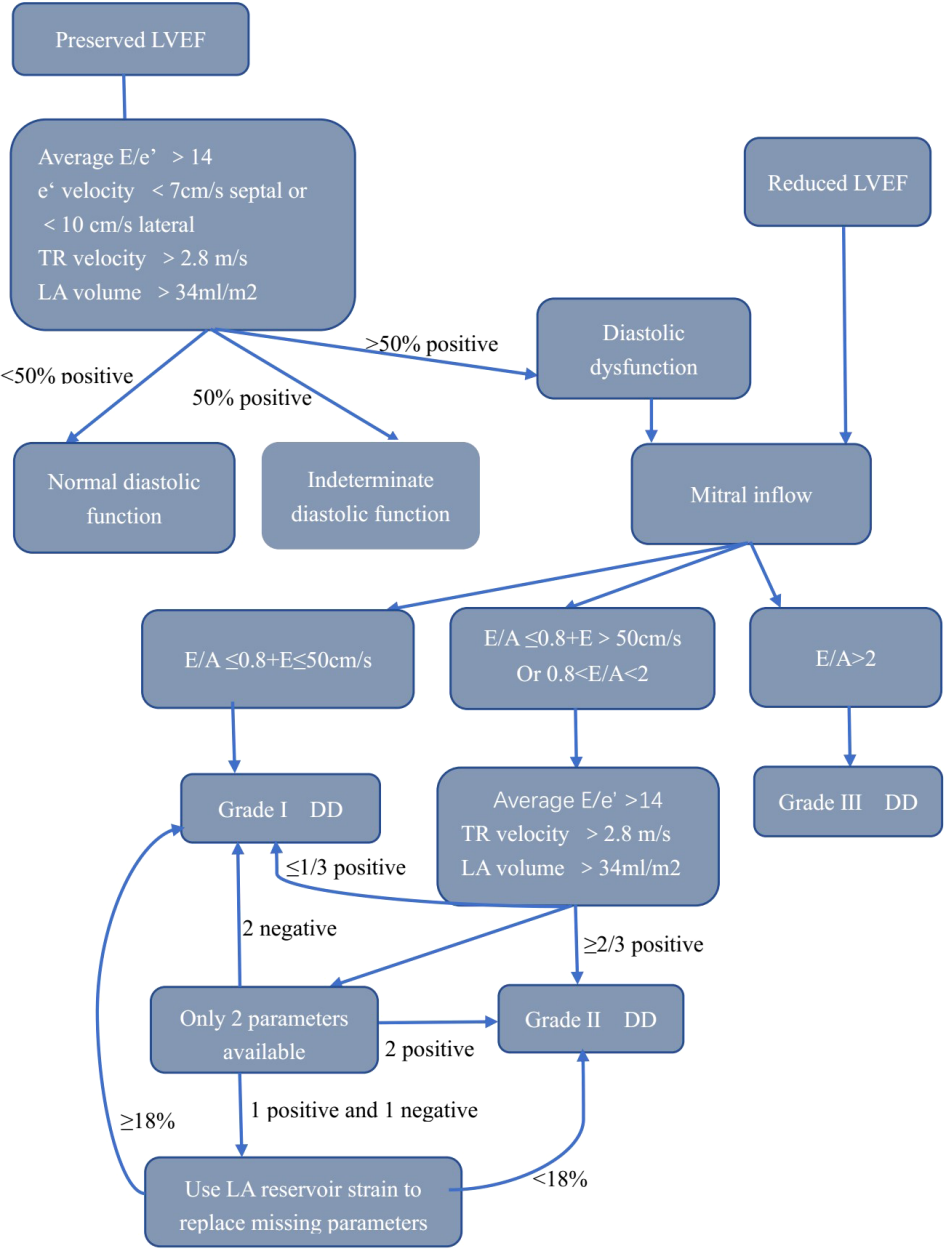
Algorithm for estimation of LVDD. LVEF, left ventricular ejection fraction; E, early filling; e’, early diastolic; LA, left atrium; TR, tricuspid regurgitation; LVDD, left ventricular diastolic dysfunction.

LVDD is primarily assessed using echocardiography. The methodology is the most extensively validated, and it has the highest temporal resolution. We have summarized the main advantages, disadvantages, and indications for utilizing different echo parameters in **Table 2**. LVDD is widely evaluated with echocardiography due to its availability and relative low cost. The first recommendations for LVDD by echocardiography were published in 2009 (7). In patients with abnormal diastolic function, the severity of LVDD was determined by comparing the maximum diastolic velocity of the early(E) and late(A) waves, E/A ratios, E-wave deceleration time, diastolic velocities (tissue Doppler) in the mitral annulus (e’ and a’), and pulmonary vein inflow. More recently, updated recommendations from the European Association of Cardiovascular Imaging (EACVI) and the American Society of Echocardiography (ASE) for the evaluation of diastolic function by echocardiography were published in 2016 (8). These recommendations tried to apply the most feasible and reproducible measurements from the 2009 recommendations to simplify the evaluation of LVDD. The 2016 recommendations proposed a new algorithm to assess LVDD, as shown in **Figure 1**. A recent study has revealed a new LVDD indicator, left atrial (LA) reservoir strain, which has excellent feasibility of ~95% and can detect LV diastolic alterations and elevated LV filling pressure even when LA volume index (LAVI) is normal (9, 10).

**Table 2:** Echocardiographic variables for evaluating left ventricular diastolic function.

Variable	Hemodynamic determinants	Advantages	Limitations
Mitral E/A ratio	E velocity is dependent on LA-LV pressure gradient in early diastole and therefore LV relaxation and LA pressure. A-wave velocity depends on LA-LV pressure gradient during late diastole, and therefore LV stiffness and LA contractility. The mitral inflow E/A ratio is used to identify the following filling patterns: normal, impaired relaxation, pseudonormal (PN), and restrictive filling.	1. Feasible and reproducible. 2. It provides diagnostic and prognostic information. 3. In patients with dilated cardiomyopathy, filling patterns correlate better with filling pressures, functional class, and prognosis than LVEF. 4. A restrictive filling pattern in combination with LA dilation in patients with normal EFs is associated with a poor prognosis similar to a restrictive pattern in dilated cardiomyopathy.	1. The U-shaped relation with LV diastolic function makes it difficult to differentiate normal from PN filling, particularly with normal LVEF, without additional variables. 2. If mitral flow velocity at the start of atrial contraction is >20 cm/s, the E/A ratio will be reduced due to fusion. 3. Not applicable in atrial fibrillation/atrial flutter patients. 4. Age dependent (decreases with aging).
DT of mitral E velocity (ms)	In patients with impaired LV relaxation, IVRT <70 ms is usually associated with increased LA pressure	1. Feasible and reproducible. 2. A short DT in patients with reduced LVEFs indicates increased LVEDP with high accuracy both in sinus rhythm and in atrial fibrillation.	1. DT does not relate to LVEDP in normal LVEF 2. Should not be measured with E and A fusion due to potential inaccuracy. 3. Age dependent (increases with aging). 4. Not applied in atrial flutter.
IVRT (ms)		1. Overall feasible and reproducible. 2. IVRT can be combined with other mitral inflow parameters, such as the E/A ratio, to estimate LV filling pressures in patients with HFrEF 3. It can be applied in patients with mitral stenosis in whom the same relation with LV filling pressures described above holds. 4. In patients with MR and in those after MV replacement or repair, it can be combined with E/e’ to estimate LV filling pressures	1. IVRT duration is in part affected by heart rate and arterial pressure. 2. It is more challenging to measure and interpret with tachycardia. 3. Results differ on the basis of using CW or PW Doppler for acquisition.
Pulmonary vein systolic-to-diastolic (S/D) velocity ratio	S/D is inversely related to LA pressure and is most reliable in patients without mitral valve disease and with depressed LVEF.	1. Reduced S velocity, S/D ratio < 1, and systolic filling fraction. 2. In patients with AF, DT of diastolic velocity (D) in pulmonary vein flow can be used to estimate mean PCWP.	1. The feasibility of recording PV inflow can be suboptimal, particularly in ICU patients. 2. The relationship between PV systolic filling fraction and LAP has limited accuracy in patients with normal LVEF, AF, mitral valve disease, and HCM.
Pulmonary vein atrial reversal duration minus mitral A velocity duration (Ar-A) (ms)	In patients with normal LA systolic function, the time difference between the duration of pulmonary vein flow and mitral inflow during atrial contraction is directly related to LV pressure rise with LA contraction and LV end-diastolic pressure.	1. PV Ar duration > mitral A duration by 30 msec indicates an increased LVEDP. 2. Independent of age and LVEF. 3. Accurate in patients with MR and patients with HCM.	1. Adequate recordings of Ar duration may not be feasible by TTE in several patients. 2. Not applicable in AF patients. 3. Difficult to interpret in patients with sinus tachycardia or first-degree AV block with E and A fusion
LA maximum volume index	LA volume is directly but weakly related to LV filling pressure.	1. Feasible and reproducible. 2. It provides diagnostic and prognostic information about LV diastolic dysfunction and chronicity of disease. 3. Apical four-chamber view provides visual estimate of LA and RA size, which confirms LA is enlarged.	1. LA dilation is seen in bradycardia, high-output states, heart transplants with biatrial technique, atrial flutter/fibrillation, and significant mitral valve disease, despite normal LV diastolic function. 2. LA dilatation occurs in well-trained athletes who have bradycardia and are well hydrated. 3. Suboptimal image quality, including LA foreshortening, in technically challenging studies precludes accurate tracings. 4. It can be difficult to measure LA volumes in patients with ascending and descending aortic aneurysms as well as in patients with large interatrial septal aneurysms
Peak velocity of tricuspid regurgitation jet by continuous-wave Doppler (m/s)	In patients without pulmonary disease, there is a direct relation between pulmonary artery systolic pressure and LA pressure.	Systolic PA pressure can be used as an adjunctive parameter of mean LAP. Evidence of pulmonary hypertension has prognostic implications.	1. Indirect estimate of LAP. 2. Adequate recording of a full envelope is not always possible, though intravenous agitated saline or contrast increases yield. 3. With severe TR and low systolic RV-RA pressure gradient, the accuracy of calculation is dependent on a reliable estimation of systolic RA.
e’ (cm/s), acquired by pulse tissue Doppler (recommended to measure at septal and lateral annulus)	The hemodynamic determinants of e’ velocity is LV relaxation, restoring forces, and filling pressure.	1. Feasible and reproducible. 2. LV filling pressures have a minimal effect on e’ in the presence of impaired LV relaxation. 3. Less load dependent than conventional blood-pool Doppler parameters.	1. Limited accuracy in patients with CAD and regional dysfunction in the sampled segments, significant MAC, surgical rings or prosthetic mitral valves and pericardial disease. 2. Need to sample at least two sites with precise location and adequate size of sample volume. 3. Different cut-off values depending on the sampling site for measurement. 4. Age dependent (decreases with aging).
Mitral E/e’ ratio	e’ is dependent on LV relaxation. As e’ corrects for the effect of LV relaxation on E, the E/e’ ratio relates directly to LA pressure.	1. Feasible and reproducible. 2. Values for average E/e’ ratio < 8 usually indicate normal LV filling pressures, values > 14 have high specificity for increased LV filling pressures.	1. E/e’ ratio is not accurate in normal subjects, patients with heavy annular calcification, mitral valve and pericardial disease. 2. ‘‘Gray zone’’ of values in which LV filling pressures are indeterminate. 3. Accuracy is reduced in patients with CAD and regional dysfunction at the sampled segments. 4. Different cut-off values depending on the site used for measurement.
LA reservoir strain	Reflects LA reservoir function and is related inversely to LA pressure. In conjunction with LA pressure, it can be used as an index of LA stiffness.	1. Feasible and reproducible 2. LA reservoir strain may be used as additional markers of LV filling pressure.	1. Lower accuracy with tachycardia as it relates to frame rate. 2. Limited data on its accuracy in the presence of atrial arrhythmias, mitral valve disease, and MAC; dependent on LV systolic function, which, if reduced, can be associated with reduced LA reservoir strain in the presence of normal LA pressure.

LVEF, left ventricular ejection fraction; LA, left atrium; LV, left ventricular; E, early filling; DT, E peak deceleration time; LVEDP, left ventricular end-diastolic pressure; MR, mitral regurgitation; MV, mitral valve; CW, continual wave Doppler; PW, pulse wave Doppler; PCWP, pulmonary capillary wedge pressure; PV, pulmonary vein; LAP, left atrium pressure; HCM, hypertrophic cardiomyopathy; RA, right atrium; ICU, intensive care unit; AV, atrium ventricular; e’, early diastolic; IVRT, isovolumic relaxation time; MV, mitral valve; TR, tricuspid regurgitation; CAD, coronary artery disease; MAC, mitral annular calcification; LVDD, left ventricular diastolic dysfunction.

## 4 Hypertension and LVDD

A link between LVDD and hypertension was found (11), in which insulin resistance, LV concentric remodeling/hypertrophy (12), abnormalities of the renin–angiotensin–aldosterone system (RAAS) (13), endothelial dysfunction, and changes of coronary microcirculation (14) were factors. High sodium intake may affect BP parameters and arterial wall damage in hypertensive individuals, contributing to LVDD impairment (15). Sympathetic nerve system (SNS) activity causes LVDD in hypertension patients, and sympathoinhibition prevents the development or delays the progression of LVDD. Diastolic dysfunction was presumed to be a surrogate marker of myocardial fibrosis in hypertensive patients who were untreated. Matrix metalloproteinases type I (TIMP-1) tissue inhibitors correlate with diastolic dysfunction (16).

LVDD can occur across a range of blood pressure states, including normal blood pressure, hypertension, or masked hypertension. Grade 1 DD was more frequent in subjects with prehypertension, and grade 2 DD was significantly frequent in hypertension. Asymptomatic and newly diagnosed hypertensive patients showed reduced E/A and lower e’ velocity compared to normal BP individuals. Inter-visit systolic blood pressure (SBP) variability was more correlated with LVDD than mean SBP. Hypertensive patients have high pulse pressure and arterial stiffness, with significant effects on LVDD through ventricle-arterial coupling. Using the 2009 recommendations, Grade 1 DD was documented in 24.4%, grade 2 DD in 19.3%, and no patients were diagnosed with grade 3 LVDD. After applying the 2016 recommendations, LVDD was documented in 12.3% of patients (17). The prevalence of LVDD is 1.2%–2.7% in adults. An early stage of impaired glucose metabolism and diabetes in hypertensive patients may specifically deteriorate diastolic function. Obesity-related insulin resistance amplifies the effect of hypertension on LVDD. In elderly hypertensive women, LVDD occurs earlier and estimated filling pressures are higher, which indicates a greater likelihood of HFpEF. Primary hypertension patients experience a decrease in LA strain conduit and reservoir function before the diagnosis of LVDD is established.

Due to their ability to reduce both preload and afterload, angiotensin-converting enzyme inhibitors (ACEIs) and angiotensin-II receptor blockers (ARBs) are conceptually the most effective treatments for LVDD. Furthermore, these drugs reverse the concentric geometry of the left ventricle and reduce myocardial fibrosis and LV hypertrophy (LVH). Recent studies have examined the clinical effect of ARBs on LVDD. The E/e′ ratio of uncomplicated hypertensives was reduced by irbesartan (18). Angiotensin receptor-neprilysin inhibitors (ARNI) are recommended for the treatment of HFpEF. In addition to LVH regression, telmisartan therapy induced a parallel decrease in LAV and a shortening of IVRT (19). LV filling time is prolonged when the heart rate is lower, which counterbalances the resistance of a stiffened left ventricle to the diastolic inflow. In hypertension patients with LVDD, controlling heart rate is therefore an important objective. There was a strong correlation between e′ velocity and the extent of SBP reduction (SBP target <130 mm Hg or <140 mm Hg), and patients with the lowest achieved SBP values tended to have the highest velocity (20).

## 5 Diabetes and glucose intolerance and LVDD

Even patients with well-controlled diabetes without overt macrovascular complications, LV structural changes, and systolic and diastolic dysfunction have all been observed. It may be triggered by insulin resistance, abnormal substrate utilization by the myocardium, and uncoupling of mitochondrial oxidative phosphorylation. Researchers have identified decreased cardiomyocyte function in diabetic animal models, caused by impaired mitochondrial calcium handling and decreased levels of free matrix calcium, as a key mediator of heart failure. Inflammatory signaling and collagen metabolism are also affected by hyperglycemia via oxidative stress, protein kinase C activation, and advanced glycosylation end-products (AGEs). Additionally, insulin resistance may contribute to SNS activity. As a result of SNS activity, the RAAS may be stimulated and may promote LVDD via adrenergic-mediated hypertrophy and fibroblast growth, as well as apoptosis and necrosis in myocytes. A high level of insulin resistance, hyperglycemia, and increased metabolism of free fatty acids (FFAs) may contribute to diabetes-related altered cardiac phenotypes (21). Diabetic patients who had microvascular complications were most likely to develop cardiomyopathy, and several of them displayed alterations within the coronary arteries of the myocardium. Diabetic hearts in humans also display thickened capillary basement membranes and capillary microaneurysms (22). In a study by Zoneraich et al. (23) in type 1 diabetic patients with normotensive blood pressure levels, small vessel disease was present in 72%, but it was not found in non-diabetic patients with normal blood pressure levels.

Glucose interacts with collagen to form AGEs, and in diabetes, the process is accelerated, leading to interstitial fibrosis. In chronic hyperglycemia, vascular and membrane proteins are non-enzymatically glycated, resulting in reactive oxygen species (ROS) and AGEs. As a result of diabetes, AGEs of collagen are formed more frequently in the myocardium of the heart, which causes stiffness. Furthermore, glucose enhances the production of the extracellular matrix in fibroblasts by activating the collagen gene promoter sequence and increasing the level of angiotensin II type 1 receptors (24). The RAAS and SNS are major neurohormonal systems that affect cardiac remodeling; therefore, drugs that affect them are vital in preventing or reversing it (24). Diabetes-related changes in the heart include hypertrophy of myocytes, the addition of extracellular collagen, interstitial fibrosis, and microangiopathy within the myocardium (25).

In individuals with type 1 or 2 diabetes, LVDD is considered to be the first manifestation of diabetic cardiomyopathy, especially in patients with poorer glycemic control. In the Strong Heart Study, which enrolled 2,411 Native Americans, type 2 diabetes was frequently associated with an abnormal LV relaxation pattern, independent of age, BP, LV mass, and LV systolic function. There is a greater degree of abnormal LV relaxation in the diabetes-hypertension combination. HbA1c concentrations correlate with abnormal LV relaxation (26). In the context of LVDD, microalbuminuria is an independent risk factor, perhaps as a marker for intramyocardial microangiopathy. It appears that LVDD is more commonly associated with aging and chronic diabetes in patients with well-controlled diabetes than hypertension or LV hypertrophy. Diabetes patients’ E/A ratios in their 40s were not significantly different from those of control subjects, but in their 50s, 60s, and 70s, the ratio was significantly lower. Diabetes patients without overt heart disease are more susceptible to diastolic function deterioration when they are older, have retinopathy, and have increased BP over time (27). Diabetes duration was strongly and positively associated with larger LAVI. Worsening in e’ (5.4 vs. 7.3 cm/s) and E/e’ ratio (13.6 vs. 10.3) was observed in patients with diabetes or impaired glucose tolerance who also had cardiovascular autonomic neuropathy (28). Impaired LV longitudinal systolic and diastolic strains were documented in diabetes. One study found the diabetes susceptibility locus, HNF1B, is associated with prevalent diastolic dysfunction and incident cardiovascular disease. This may provide future drug targets (29). Those with a higher HbA1c level and obesity status showed a high prevalence of LVDD (30). Decreased LA reservoir, conduit, and booster strain was found in adolescents and young adults with obesity and diabetes, although LA volume was normal (31). Peak diastolic strain rate from cardiovascular magnetic resonance feature tracking was decreased in diabetes (32).

Glycemic control can partially prevent or reverse LVDD. Canagliflozin can improve LV diastolic function within 3 months, and those with significantly improved hemoglobin values showed the greatest benefit (33). Animal research found that PPAR-α (fenofibrate) or -γ agonists (pioglitazone) prevented LVDD, possibly by improving the fatty acid metabolism in the myocardium or by modifying hyperglycemia and/or hyperlipidemia (34). After 4 years of treatment with pioglitazone on LVDD in diabetes, a study found an increase in both E/e’ and LAVI (35). Liraglutide therapy had favorable effects on E/e’ regardless of body weight. The DPP-4 inhibitor, alogliptin, prevents cardiac diastolic dysfunction by inhibiting ventricular remodeling, which can be explained by enhanced mitochondrial function and increased mitochondrial biogenesis in diabetic rabbits (87). LVEF and E/e’ in individuals with diabetes improved after 6 months of treatment with tofogliflozin, a sodium-dependent glucose transporter 2 (SGLT2) inhibitor. A previous study has indicated that empagliflozin improves diastolic function, preserves calcium handling and growth signaling pathways, and reduces myocardial insulin resistance in obese mice (36). SGLT2 inhibitors (SGLT2is) are efficacious and safe in treating HFpEF in patients with comorbid chronic kidney disease with and without T2DM (37). SGLT2is work by inhibiting the sodium-glucose co-transporter 2 in the proximal renal tubules, reducing glucose reabsorption and increasing glucose excretion in urine. This leads to lower blood glucose levels, improved insulin sensitivity, and additional benefits such as reduced sodium retention, blood pressure, and cardiac workload, which are particularly beneficial in managing heart failure and diabetic conditions. The ESC 2023 HF guideline update has given SGLT2is for chronic HFpEF a class 1a recommendation (38). However, in patients with diabetes and hypertension without overt heart failure, metformin treatment did not affect LV mass or diastolic function after 1 year (39).

## 6 Obesity and LVDD

Previous histological analysis showed that the capillary length density of obese patients was lower (40), cardiomyocyte width was reduced, and the pulmonary capillary wedge pressure was greater. A reduction in phosphocreatine/adenosine triphosphate (ATP) is further exacerbated in obese individuals during inotropic stress, leading to continuing diastolic dysfunction. There is evidence that myocardial energetics may play a key role in obesity-related diastolic dysfunction. The obesity-related increase in LV filling pressure was associated with a lower coronary microvascular density, which could account for the lower maximal myocardial blood flow, impaired myocardial metabolism impairment, LVDD, and a greater risk of HF in obese individuals. Excess adipose accumulation in peripheral obesity increases total and central blood volume, resulting in an increase in cardiac output. Peripheral vascular resistance is decreased, which facilitates this process. An increased amount of visceral adipose tissue is associated with low-grade inflammation (serum C-reactive protein) (41). Obesity is associated with various neurohormonal and metabolic abnormalities that may alter cardiac morphology in humans. Obesity is associated with LVH (42), which alters cardiac morphology. The combination of insulin resistance and hyperinsulinemia has been linked to increased LV mass in obese animals and humans. LVH contributes the most to LV diastolic function. In obesity, RAAS activation may increase sympathetic tone and directly affect the LV myocardium, which can lead to LVH (43). LVDD and myocardial fibrosis were also exacerbated by obesity and hypertension in a synergistic manner (44). It is often difficult to determine whether obesity is independently responsible for LVDD in obese patients due to the presence of insulin resistance, impaired glucose tolerance, or overt diabetes (43).

Impairment of LV diastolic filling or relaxation in obesity was revealed. LVDD appears to be related to obesity level and fasting insulin levels and reduced exercise capacity (45, 46), however, obesity duration was not considered (47). Elderly patients with severe prolonged obesity had elevated plasma volume, eccentric LVH, and systolic and diastolic dysfunction. HF is more likely to develop in obese women. The adverse effect of central adiposity on LV diastolic function was independent of general adiposity and more prominent among women (48). Only visceral fat, other than total body fat, was significantly associated with LVDD. The relationship between LV diastolic function and visceral fat was significantly mediated by triglycerides and sex hormone-binding globulin, possibly through a metabolic pathway that involves blood lipids and ectopic fat accumulation (49). A significant association exists between adipocyte fatty acid-binding protein (FABP4) levels and LVDD in obese subjects who have MetS. FABP4 may thus play a role in obesity and cardiometabolic disorders (50). In morbidly obese individuals, the growth-differentiation factor (GDF)-15 level, a marker of inflammation, was better correlated with diastolic dysfunction. GDF-15 levels increase in relation to different degrees of LVDD (51). One study showed obese patients with a reduced diffusing capacity of the lungs for carbon monoxide had an increased prevalence of moderate or severe LVDD (52). Sarcopenic obesity was associated with impaired diastolic function and decreased exercise capacity (53, 54).

Weight loss, whether achieved through diet and exercise or bariatric surgery, can improve myocardial metabolism, left ventricular structure, and diastolic function, all of which are affected by obesity. As BMI decreased longitudinally, LAVI decreased significantly, and e’ velocity increased significantly. Surgical weight loss resulted in a 23% decrease in LV mass, a 33% increase in E/e, and a 28% improvement in relaxation. A reduction of BMI, insulin resistance, total oxygen consumption of the heart, and LV mass was associated with an improvement in LV relaxation but not the reduction of fatty acid utilization. These changes can be reversed more effectively by bariatric surgery than by diet and exercise alone. Bariatric surgery, which is primarily used for severely obese patients, results in significant weight loss and improves the neurohormonal and metabolic milieu to a greater extent than the weight loss modalities of diet and exercise alone (55).

High- and moderate-intensity training can prevent diet-induced obesity-related LV remodeling with diastolic and systolic dysfunction in mice, which suggests that physical activity can alleviate obesity-related cardiac disorders. An obese, insulin resistance, and hypertension rodent model showed that nebivolol attenuated diastolic dysfunction and myocardial remodeling by blunting myocardial oxidative stress and promoting insulin metabolic signaling (56). Recent evidence indicates that GLP-1 RA may play a significant role in preventing HFpEF in patients with obesity, MS, or obesity and T2DM (57). According to a recent study, Epithelial Sodium Channel (EnNaC) activation induces endothelium permeability, which promotes macrophage infiltration and oxidative stress, resulting in cardiac fibrosis and LVDD in female mice with diet-induced obesity. Western diet-induced impairments of left ventricular filling rate and relaxation time were attenuated by amiloride, an EnNaC antagonist (58). A significant reduction in cardiomyocyte area, interstitial and perivascular fibrosis, and collagen deposition was observed after clostazol treatment. The inflammatory milieu in the hearts of obese mice was also reduced by cilostazol. There may be a therapeutic role for clostazol in treating obesity-related diastolic dysfunction and preventing overt heart failure (59).

## 7 Hyperlipidemia and LVDD

There is an association between LVDD and increased myocardial lipid storage. Diastolic dysfunction can be induced by lipid intermediates, which generate ROS. Mitochondrial dysfunction and impaired energetics may lead to cardiac dysfunction since the heart requires most of its ATP from mitochondrial oxidative phosphorylation. Reduced ATP availability may be caused by mitochondrial dysfunction but could also be caused by myocardial lipid buildup. It has been discussed previously that mitochondrial dysfunction leads to an increase in mitochondrial ROS, which damages cellular components and causes subsequent dysfunction (60). Increased oxidative stress and inflammation in a high-fat and high-cholesterol diet in rats led to cardiac fibrosis, endothelial dysfunction, and LVDD (61).

Intramyocardial fat deposition may partly contribute to LVH and impaired diastolic function in humans. When all components of the MetS and visceral adiposity tissue were adjusted for, an increase in hepatic triglyceride content was associated with a change in mean E/A in obese individuals. In healthy subjects or those with diabetes, a short-term very low-calorie diet (VLCD) induced the accumulation of myocardial triglycerides and was associated with a decrease in LV diastolic function (62, 63). An increase in intramyocardial triglycerides is associated with LVDD. Myocardial steatosis is associated with accelerated deterioration of left ventricular diastolic function over time. In children with heterozygous familial hypercholesterolemia (FH), reduced e’ and higher E/e’ ratios were observed (64). In one Mendelian randomization (MR) analysis, HDL cholesterol showed no significant connection with any LV parameter. LDL cholesterol and triglycerides were independently associated with adverse changes in LV mass in another MR analysis (65).

There is controversy regarding the effect of lipid-lowering therapy on improving diastolic function. Compared with healthy volunteers, patients with hypercholesterolemia showed lower E/A ratios, higher Tei indexes, and lower e’/a’ ratio in both the septum and laterally. After 6 months of rosuvastatin (RSV) treatment, a significant improvement of longitudinal global systolic and diastolic function (Tei index) was registered (66). The subjects received treatment with RSV or pitavastatin (PTV) for 24 weeks, however, the result showed statin treatment did not significantly alter the E/e′ ratio (67). Multiple diastolic parameters should be included in this research to avoid bias. In rabbits, HDL cholesterol infusions accompany a rapid improvement of LVDD with reductions of LV macrophage accumulation, coronary atherosclerosis, cardiomyocyte apoptosis, and remodeling (68).

## 8 MetS and LVDD

Although the effect of the MetS on LV remodeling has been extensively studied, the effect of MetS on LVDD has been less studied (69–73) (**Table 3**). Previous studies have revealed that patients with MetS have LVDD independent of LV mass (74, 75). In patients with MS, insulin resistance plays a key role in LVDD and HFpEF (76). Additionally, SNS excitement, RAAS activation, oxidative stress, endothelial dysfunction, and inflammation, which are common symptoms of MetS, could also explain the worsening of LVDD in these patients. In MetS, excess salt induces LVDD through the upregulation of mineralocorticoid receptor signals and increased oxidative stress. Myocardial fibrosis is a key contributor to subclinical LVDD and HFpEF in patients with MetS. In patients with MetS, those with increased abdominal fat deposition exhibited higher levels of procollagen peptides and cardiac fibrosis and more severe LVDD manifested by lower myocardial remodeling and e’ and higher E/e’ ratios (77). MetS and higher insulin resistance were significantly related to impaired diastolic function in one study using cardiac magnetic resonance imaging (CMR), independent of the myocardial extracellular matrix (ECV). The severity of LVDD was strongly related to the abundance of MetS components. Importantly, diabetes and obesity affect LV function even in the absence of coronary artery disease and hypertension (78). LVDD was more pronounced in individuals with obesity and MS than in those without (79). LVDD can also occur in patients with MetS without hypertension. Impaired LV diastolic and systolic synchronization were found in patients with MS, in which obesity, hyperglycemia, and age play key roles, whereas hypertension was not a contributor to impaired synchronicity. A previous study has shown that the impact of the MetS on preclinical myocardial abnormalities were not accounted for by differences in age, gender, or 24-h BP and can be reasonably ascribed to the interplay of MetS components, making MetS in itself a relevant clinical problem in non-diabetic patients or in those never treated with antihypertensive or lipid-lowering drugs (80). Additionally, the coexistence of MS with hypertension or diabetes can further worsen LVDD (81, 82).

**Table 3:** The impact of MetS on LVDD.

Reference	Study population (n)		e’ cm/s		E/e’		LAVI		E/A		LVDD	
	MS+	MS-	MS+	MS-	MS+	MS-	MS+	MS-	MS+	MS-	MS+	MS-
Lisa de las Fuentes (74)	186	110	9.0 ± 2.1	11.5 ± 2.4*	–	–	–	–	1.2 ± 0.5	1.6 ± 0.5*	7-9%	29-35%
Nir Ayalon (91)	90	26	9.0 ± 2.0	11.7 ± 3.0*	9.2 ± 2.4	6.6 ± 1.7*	–	–	1.1 ± 0.3	1.6 ± 0.5*	–	–
Parvanescu, T (69).	150	150	–	–	–	–	–	–	0.81 ± 0.21	1.47 ± 0.23*	52%	39%*
Chung, J. W (70).	20	17	4.7 ± 0.8	5.5 ± 1.5*	–	–	32.8 ± 6.8	45.1 ± 20.0*	0.55 ± 0.09	0.67 ± 0.17*	–	–
Jorgensen,P.G (71).	345	80	6.5 ± 1.8	8.4 ± 2.7*	11.6(9.6, 14.9)	7.7 (6.6, 9.9) *	26 (22, 32)	25 (20, 31) *	0.87(0.75, 1.06)	1.11(0.85,1.38) *	–	–
Burroughs Pena, M (72).	399	861	6.8 ± 0.1	7.5 ± 0.1*	10.0 ± 0.2	9.3 ± 0.2*	22.7 ± 0.5	23.2± 0.03*	1.0± 0.02	1.1 ± 0.01*	–	–
Aksoy, S (81).	30	30	7.3 ± 0.4	14 ± 3*	8.9 ± 2.2	6.6 ± 1.3*	31.5 ± 9	23.1 ± 9.4*	0.9 ± 0.3	1.4 ± 0.1*	–	–
Crendal, E (73).	92	50	8.7 ± 1.5	10.6 ± 1.8*	6.9 ± 1.9	5.7 ± 1.5*	–	–	1.1 ± 0.3	1.3 ± 0.4*	–	–
Kosmala, W (77).	172	61	6.0 + 1.9	9.4 + 2.5*	10.9 + 3.3	8.1 + 2.4*	–	–	1.17 + 0.42	1.38 + 0.44*	–	–
Hwang, Y.-C (92).	331	1228	6.2 ± 1.5	7.4 ± 1.8*	9.7 ± 2.5	8.4 ± 2.0*	21.9 ± 5.5	20.2 ± 5.8*	0.90 ± 0.25	1.09 ± 0.34*	–	–

MetS, metabolic syndrome; LVDD, left ventricular diastolic dysfunction.

* Subjects with metabolic syndrome vs. control group (p< 0.05).

A previous study confirmed a significant improvement in LVDD after a 1-year lifestyle intervention program in abdominally obese men with MS, which worked through improved exercise tolerance, enhanced heart rate variability (HRV), and decreased insulin levels (83). One study showed that adding spironolactone to standard angiotensin II inhibition therapy significantly improved myocardial fibrosis in patients with MS (84). In a rat model of metabolic syndrome, improving endothelial dysfunction could improve LVH and diastolic function (85, 86). The DPP-4 inhibitor linagliptin reduces left ventricular stiffness and improves LV relaxation, which was related to decreased cardiac fibrosis and cardiomyocyte passive stiffness in MetS rats (87). In MetS rats, calorie restriction alleviates the incidence of obesity, hypertension, LV remodeling, and diastolic dysfunction by reducing cardiac oxidative stress and inflammation (88). Statins treatment reverses myocardial remodeling and enhances ventricular relaxation via AMPK (amplifier-activated protein kinase)-mediated antifibrotic effects (89). Attenuation of the inflammatory and oxidative stress process, reduced insulin levels, and decreased cardiac fibrosis may provide a novel therapeutic strategy in treating metabolic cardiomyopathy.

## 9 Clinical consequences of LVDD in individuals with metabolic syndrome

LVDD is an independent predictor of adverse cardiovascular events in hypertensive patients. Of importance, the prognostic value of e′ and the E/e’ ratio for heart failure has been recognized in the hypertensive setting with normal EF. The Framingham Heart Study also provided the earliest evidence of diabetes and heart failure independent of coronary artery disease (90). In individuals with overt cardiovascular diseases, MetS increases the risk of adverse events; however, in HF patients, MetS was not an independent predictor for all-cause mortality or cardiovascular mortality. Few studies have investigated the prognostic value of LVDD in patients with MetS. The coexistence of MetS with diastolic dysfunction showed obvious incremental value in predicting cardiac events. Overall, few prospective studies have explored the association between LVDD and HFpEF in patients with MetS. Much of the current evidence is based on the characterization of patients with HFpEF. MetS refers to a group of interrelated disorders. More studies have explored the prognosis of LVDD in patients with hypertension or diabetes. Along with an aging population and rising rates of cardiometabolic comorbidities, HFpEF has a tremendous global burden and is poised to increase in prevalence, particularly the MetS phenotype. Future research should focus on the prognostic value of LVDD in patients with metabolic syndrome. A previous study revealed that the number of MetS criteria fulfilled and the presence of 4–5 criteria was associated with incident HF, implying that metabolic disturbances also contribute to an elevated risk of HF through pathways other than insulin resistance (76).

## 10 Conclusion

**Figure 2:**
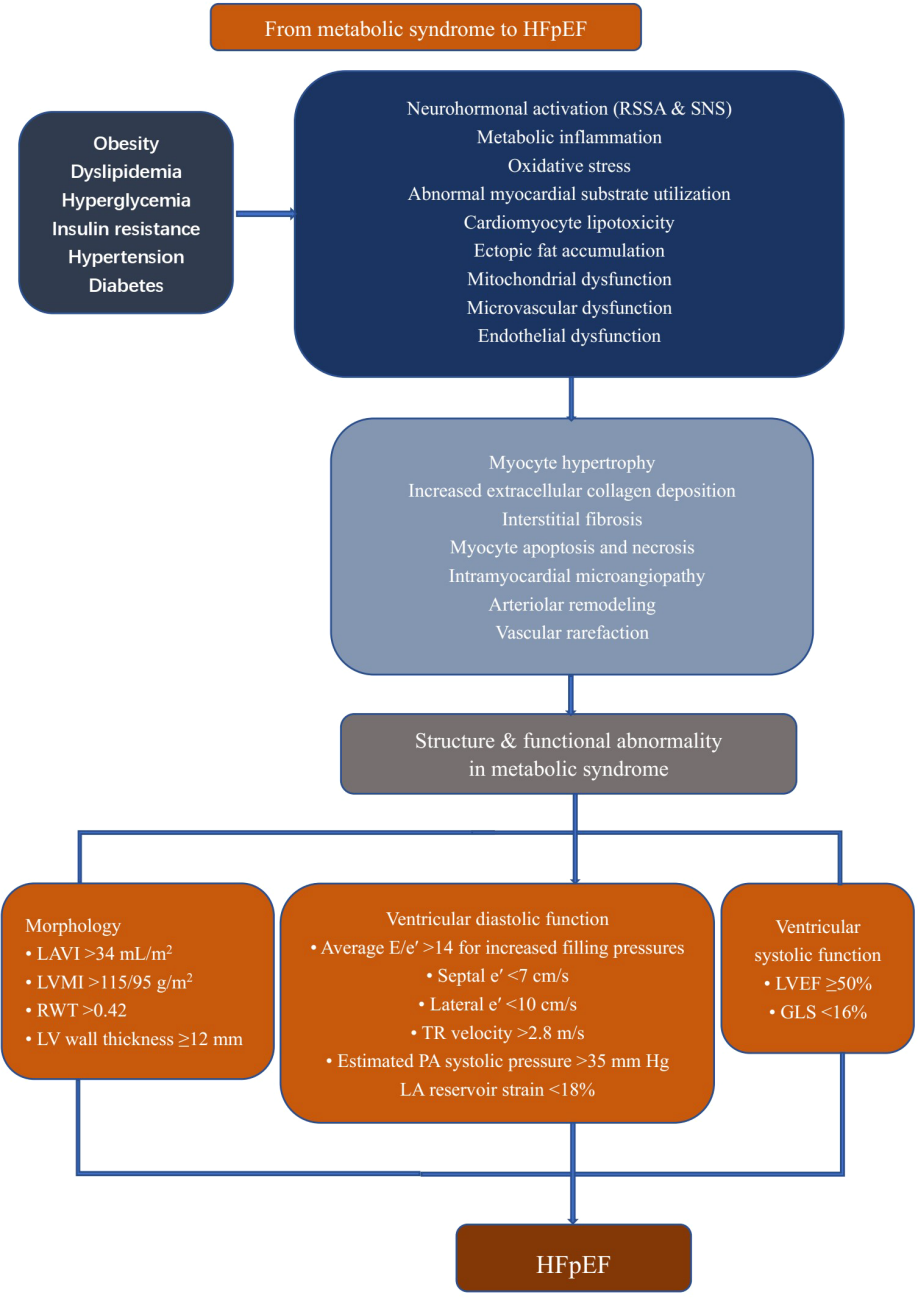
Cardiac remodeling in patients with metabolic syndrome. All components of metabolic syndrome can induce cardiac alteration to some extent. Their sum activates several pathophysiological pathways in the vessels and heart, including neurohormonal activation, mitochondrial dysfunction, and increased oxidative and inflammatory stress, leading to adverse cardiac remodeling. LVEF, left ventricular ejection fraction; E, early filling; e’, early diastolic; LA, left atrium; LAVI, left atrium volume index; LVMI, left ventricular mass index; RWT, relative wall thickness; TR, tricuspid regurgitation; PA, pulmonary artery; GLS, Global Longitudinal Strain; HFpEF, heart failure with preserved ejection fraction; RSSA, Renin Angiotensin Aldosterone System; SNS, sympathetic nervous system.

The prevalence of MetS is constantly growing. MetS-related LVDD or HFpEF also leads to a high risk of cardiac events in this population. Echocardiography diagnosis techniques are rapidly developing, allowing for the early detection of cardiac structural or functional alterations, as summarized in **Figure 2**. Although the underlying mechanisms of HFpEF pathogenesis remain controversial, insulin resistance and subsequent changes in both cardiomyocytes and the myocardial interstitium and coronary microcirculation play critical roles in mediating LVDD in MetS. Considering this, in individuals with MetS, improving LVDD may serve as the therapeutic strategy to prevent or ameliorate HFpEF, especially for the MetS phenotype. Improving metabolic abnormalities associated with MetS, particularly through controlling insulin levels and reducing oxidative stress and inflammation, may help slow down or reverse the progression of LVDD, thereby improving the prognosis of HFpEF. Although lifestyle interventions (such as weight loss and exercise) and pharmacological treatments (such as statins and liraglutide) have been shown to have certain effects on improving LVDD, there is significant individual variability among patients with MetS, and the universality and long-term effects of these interventions are still unclear. The lack of standardized treatment guidelines and evidence-based support makes it difficult to implement clinical treatments broadly. With the continuous development of personalized medicine, future research should focus more on how to tailor treatment plans based on different MetS subtypes and patient characteristics (such as gender, age, and comorbidities). Further prospective large population studies are needed to integrate clinical data and optimize intervention strategies to enhance treatment outcomes.
